# Defensins versus pathogens: an unfolding story

**DOI:** 10.18632/oncotarget.5109

**Published:** 2015-08-06

**Authors:** Elena Kudryashova, Wuyuan Lu, Dmitri S. Kudryashov

**Affiliations:** Department of Chemistry and Biochemistry, The Ohio State University, Columbus, OH, USA

**Keywords:** Innate immunity, antimicrobial peptides, Immunology and Microbiology Section, Immune response, Immunity

Bacteria are one of the major factors shaping the evolutionary landscape of all eukaryotic organisms including humans. Bacterial epidemics, which have taken hundreds of millions of human lives in the observable history, are arguably some of the most vivid and cruel manifestations of natural selection. The history of co-evolution of eukaryotic and prokaryotic organisms is ingrained in their organization as the immune system from one side, and effector molecules supremely tuned to manipulate the essential pathways and cascades (e.g. toxins) from the other.

Bacterial proteinacious toxins are the deadliest compounds on our planet. Given the outstanding killing efficiency of toxins, eukaryotes had no choice but to develop mechanisms of their effective neutralization. The adaptive immune system does so by producing highly specific and potent neutralizing antibodies. However, 5-7 days required to produce antibody is too long to protect from immediate threats. Innate immunity, in contrast, acts promptly by recognizing molecular patterns of pathogens (i.e. characteristic and essential microbial traits: lipid A of LPS, as well as bacterial flagellin, lipoproteins, and DNA) and exerting prominent cellular and humoral response towards their sources. But how can innate immunity recognize and protect from toxic bacterial proteins, which are plentiful and diverse?

A decade ago, a group of antimicrobial effector peptides called defensins was reported to efficiently neutralize anthrax toxin both *in vitro* and in an animal model [1]. Human defensins are produced primarily by either neutrophils and intestinal Paneth cells (α-defensins) or various epithelial cells (β-defensins). The anti-toxin potential of defensins long remained hidden in the shadow of their other antimicrobial potentials - the immune-modulating abilities as well as the ability to kill microbes via disintegration of bacterial membranes, assisting in formation of neutrophil extracellular traps (NETs), and self-assembled nanonets [2]. In the years following the discovery of defensins’ ability to neutralize anthrax toxin, many other toxins were recognized as defensins’ targets. Six conserved cysteine residues connected by three characteristic disulfide bonds, several cationic and hydrophobic amino acids, as well as the ability to form dimers and/or higher order oligomers were all identified as parameters essential for toxin inactivation by these peptides [3]. Surprisingly, most of the affected toxins do not share common mechanisms of toxicity and belong to different enzymatic and non-enzymatic groups of toxins with little to no sequential or structural similarity. Given that human defensins represent a small structurally conserved group of antimicrobial peptides with limited variability, their ability to target a broad group of largely unrelated toxins remained puzzling until recently.

To address this problem, we hypothesized that all of the affected toxins are targeted based on some common property. What is this elusive property that must be essential for toxicity, or it would be relinquished for the sake of being unrecognized by the defense peptides? All the affected toxins can be divided into two distinct groups. The first group is pore-forming toxins (e.g. cholesterol dependent cytolysins, protective antigen of anthrax toxin) act via breaking the integrity of the cell membrane or providing an entry port for the second group of affected toxins (diphtheria toxin, *Clostridium difficile* toxin B, *Pseudomonas aeruginosa* toxin A, lethal and edema factors of anthrax toxin) acting in the cytoplasm of the host cell. One of the essential features of both groups is their high structural plasticity dictated by the necessity to undergo dramatic conformational transitions in response to mild environmental factors (e.g. acidification of endosomal compartments). The pore-forming toxins have to do so to shift from a water-soluble pre-pore to a membrane-integrated pore-forming structure; the pore-crossing toxins have to get unfolded to cross the pore. In both cases, the conformational transitions happen in the extracellular space and therefore cannot utilize free energy of ATP hydrolysis as it is done by intracellular folding-assisting molecules (chaperones). Therefore, the structural plasticity for many toxins roots in their marginal thermodynamic stability, which, we hypothesized, is targeted by human defensins.

To test the hypothesis we analyzed a diverse group of toxins, some of which were not previously recognized as defensin targets, but have been shown to be thermodynamically unstable [4]. We found that binding of several human defensins to toxins caused their local unfolding and destabilized secondary and tertiary structures, leading to increased susceptibility to proteolysis and increased solvent exposure of toxins’ hydrophobic residues. Therefore, defensin-induced toxin precipitation observed by us and other investigators is likely the result of toxin self-association via exposed hydrophobic residues. Both α- and β-defensins, as well as a synthetic defensin retrocyclin, which recapitulates a lost in evolution cyclic **03B8** -defensin present in human genome only as a pseudogene, caused destabilization of toxins, albeit to a different extent [5, 6]. None of the several tested mammalian structural and enzymatic proteins were destabilized by defensins; yet, it is conceivable that some of the mammalian proteins may be vulnerable to defensin effects, but remain protected by their intracellular compartmentalization, whereas defensins largely act in the extracellular space.

Importantly, the proposed mechanism of defensin-promoted destabilization of marginally stable proteins may extend well beyond targeting bacterial toxins as many viral effector proteins also demonstrate functionally essential thermodynamic instability. This hypothesis would explain how a single HNP-1 α-defensin peptide can intercept entry of HIV-1 into host cells at multiple steps [7]. Taken together, our recent study proposes that the difference in thermodynamic stability between host and pathogen proteins represents a novel, largely overlooked molecular pattern that is both recognized and targeted by major effector molecules of innate immunity - defensins. Although this pattern allows only relative selectivity against pathogenic effector molecules produced by bacteria and viruses, it is essential for immediate protection allowing time for the adaptive immune response to take place.
